# Temperature-Dependent Conversion of Wheat Straw by *Ganoderma lucidum*: A Chemical and Multi-Omics Analysis

**DOI:** 10.3390/jof12100730

**Published:** 2026-09-30

**Authors:** Jing Zhang, Xiaojun Liang, Lei Mao

**Affiliations:** 1 Institute of Animal Science, Ningxia Academy of Agriculture and Forestry Sciences, Yinchuan 750002, China; 18709519130@163.com (J.Z.);; 2 College of Animal Science and Technology, Ningxia University, Yinchuan 750021, China

**Keywords:** *Ganoderma lucidum*, wheat straw, solid-state fermentation, temperature, lignocellulosic degradation, multi-omics

## Abstract

Temperature critically regulates fungal lignocellulose degradation, but the mechanisms underlying straw conversion by *Ganoderma lucidum* for ruminant feed remain unclear. This study investigated biochemical and molecular responses of *G. lucidum* during solid-state fermentation of wheat straw at 20, 27, and 34 °C over 0, 2, 4, and 6 weeks using chemical, transcriptomic and metabolomic analyses. At week 6, lignin degradation reached 63.6% at 27 °C and 34 °C, exceeding 27.0% at 20 °C. Similarly, hemicellulose degradation reached 60% at 27 °C and 34 °C, exceeding 33.2% at 20 °C. In contrast, cellulose degradation showed more consistent temperature dependence, with 46.1% at 34 °C and 21.6% at 20 °C. Transcriptomics revealed temperature-dependent activation of carbohydrate-active enzymes and lignin-modifying genes, including up-regulated laccase genes, as well as temperature-specific induction of xylitol dehydrogenase at 27 °C and barwin-like endoglucanase at 34 °C. Metabolite profiles showed partial convergence between extended low-temperature and short-term high-temperature fermentation. Multi-omics integration identified a conserved negative correlation between phenylpropanoid/lignan and lipid/organic acid metabolites, alongside temperature-dependent remodeling of gene and metabolite networks. This study reveals chemical, transcriptomic, and metabolomic changes during solid-state fermentation of wheat straw by *G. lucidum*, providing mechanistic insights into temperature-regulated fungal biomass conversion.

## 1. Introduction

Wheat straw is one of the most abundant agricultural by-products worldwide, yet a substantial proportion of it is burned or left to decompose annually, leading to considerable resource waste and environmental pollution [1]. As a potential roughage resource for ruminants, the nutritional value of wheat straw is significantly constrained by its high lignin content [2]. Lignin, an aromatic polymer in the plant cell wall, is linked to cellulose and hemicellulose via ester and ether bonds, forming a compact lignocellulosic complex [3,4]. This structural barrier greatly restricts the degradation and utilization of structural carbohydrates by rumen microorganisms. Previous studies have shown that untreated wheat straw contains only 2–5% crude protein [5,6,7], and its in vitro dry matter digestibility is generally below 50% [6]. Therefore, controlling lignin removal together with the transformation and retention of structural carbohydrates represents a critical challenge in the biological conversion of wheat straw.

White-rot fungi are the only microorganisms in nature capable of complete mineralization of lignin. They accomplish this through the secretion of extracellular oxidative enzyme systems, including manganese peroxidases, laccases, and lignin peroxidases [8,9]. Among white-rot fungi, *Ganoderma lucidum* has attracted considerable attention due to its dual characteristics. On one hand, *G. lucidum* is a medicinal fungus whose metabolites, such as polysaccharides and triterpenoids, exhibit notable pharmacological activities [10,11]. Certain compounds derived from *G. lucidum* possess substantial antitumor, antioxidant, antiviral, and immunomodulatory properties [12]. On the other hand, this strain possesses a strong capacity for lignocellulose degradation, attributed to the diverse lignin-modifying and carbohydrate-active enzymes encoded in its genome [13]. Genome sequencing of *G. lucidum* has revealed that the highest expression of lignin-modifying enzymes, such as laccases and lignin-degrading heme peroxidases, and their corresponding genes occurs upon full colonization of the substrate by mycelia, while type I cellobiohydrolase is the most abundant extracellular lignocellulolytic enzyme. It has been reported that under specific solid-state fermentation conditions, a strain of *G. lucidum* achieved 58.5% lignin degradation with only 0.7% cellulose degradation [14], indicating that selective delignification can occur in certain *G. lucidum* strain–substrate systems. Moreover, solid-state fermentation of wheat straw with *Ganoderma* sp. rckk02 has been found to reduce acid detergent fiber and neutral detergent fiber while improving in vitro organic matter digestibility, estimated metabolizable energy, and dry matter intake in goats [15]. These findings demonstrate that *G. lucidum* holds considerable potential for the feed-oriented utilization of crop straw.

Temperature is one of the main environmental factors influencing fungal growth [16,17,18] and development [19,20]. Fluctuations in temperature can substantially affect the progress of solid-state fermentation by white-rot fungi and the extent of lignin degradation in straw [21]. Under high temperatures, fungi exhibit a reduced mycelial growth rate and lower biomass [21,22]. Similarly, in *Trichaptum abietinum*, heat stress applied after 25 days of incubation in liquid media decreased hyphal biomass, increased CO_2_ emissions, and significantly shifted carbon allocation toward metabolite production and unknown fractions [16]. In a comparative transcriptomic study, white-rot fungi *Trametes pubescens* and *Phlebia centrifuga* exhibited substrate-specific molecular responses when grown on wheat straw and spruce at 15 °C and 25 °C, with differential expression of genes encoding polysaccharide-degrading enzymes, transporters, and monooxygenases [20]. Further multi-omics integration has revealed that cellulose and hemicellulose degradation products participate in the synthesis of sugar alcohols, trehalose, and polysaccharides, and that enhanced carbohydrate metabolism provides precursors for triterpenoid biosynthesis, indicating a close metabolic coupling between substrate degradation and secondary metabolism in *G. lucidum* [23]. These findings highlight the species specific nature of temperature adaptation in white-rot fungi. While the lignin-degrading capacity of *G. lucidum* is well recognized, how environmental temperature regulates its selective degradation behavior remains poorly understood, and the underlying transcriptional and metabolic regulatory mechanisms have not yet been systematically characterized.

In this study, we integrate chemical composition analysis with transcriptomic and metabolomic profiling to systematically characterize temperature-dependent lignocellulose conversion by *G. lucidum* during solid-state fermentation of wheat straw at 20 °C, 27 °C, and 34 °C. Specifically, we aim to elucidate how temperature regulates the selectivity and extent of lignocellulose degradation, and to uncover the underlying transcriptional and metabolic mechanisms. Our findings establish a theoretical foundation for precise temperature control in fungal fermentation, thereby advancing the feed-oriented valorization of crop straw.

## 2. Materials and Methods

### 2.1. Fungal Strain and Inoculum Preparation

The white-rot fungal strain used in this study was *Ganoderma lucidum* (red strain), designated as G5 by our laboratory. It was maintained at the Institute of Animal Science, Ningxia Academy of Agriculture and Forestry Sciences, Ningxia, China. The procedures for strain activation and inoculum preparation were as follows. First, PDA (Oxoid Ltd., Basingstoke, Hampshire, UK) plates fully colonized by mycelia and stored at 4 °C were removed from the refrigerator and activated overnight at room temperature. The activated fungus was then aseptically transferred onto freshly prepared PDA plates and incubated in a constant temperature incubator at 26 °C with 70% relative humidity until the mycelia uniformly covered the entire agar surface. Subsequently, two agar plugs, each approximately 1.5 cm^2^ in area and fully colonized by mycelia, were aseptically inoculated onto sterilized sorghum grains and incubated under the same conditions (26 °C, 70% relative humidity) until the grain was fully colonized by fungal mycelium. The prepared solid-state inoculum was then stored at 4 °C until use in the subsequent wheat straw inoculation experiment.

### 2.2. Fungal Inoculation and Incubation

The wheat straw used in this study was obtained from Ningxia, China, and had been naturally air-dried and properly stored. It was mechanically chopped to a length of 1.5–3.0 cm using a forage chopper and packed into single opening mesh bags (50 × 80 cm; pore size, 1.5 × 1.5 mm). The bags were soaked in tap water at room temperature for 13 h, then removed and drained for 3 h. The straw was thoroughly mixed and then packed into plastic culture bags equipped with filter patches (300 g wet straw per bag), which allowed gas exchange while preventing contamination. The culture bags were sterilized in an autoclave at 121 °C for 1 h, then transferred to a clean bench and cooled to room temperature. For inoculation, sorghum grains fully colonized by *G. lucidum* mycelia were added at 10% (*w*/*w*) based on the air-dried weight of wheat straw and thoroughly mixed with the straw. The inoculated bags were statically incubated in constant temperature incubators (ZENITHAR ZP-800; Shanghai Zhixin Experimental Instrument Technology Co., Ltd., Shanghai, China) at 20 °C, 27 °C, and 34 °C for 0, 2, 4, and 6 weeks. Each treatment was prepared with three biological replicates. At each sampling time point, the corresponding culture bags were removed from the incubator and the contents were thoroughly mixed. The samples were then divided into subsamples. One portion of fresh material was dried, ground, and sieved for chemical composition analysis. Another portion was sealed and stored at −20 °C for subsequent pH determination. The remaining samples were immediately frozen in liquid nitrogen and stored at −80 °C for transcriptomic and metabolomic analyses.

### 2.3. Chemical Composition and pH Analysis

Fresh samples were dried in a forced air oven at 70 °C to a constant weight. The dried samples were then ground and passed through a 1 mm sieve to obtain a homogeneous powder for subsequent chemical analysis. Dry matter (DM) content was determined according to the Chinese national standard GB/T 6435-2014 [24]. Briefly, 2–3 g of each sample was dried at 105 °C for 4 h and then weighed. True protein (TP) content was determined using the copper sulfate precipitation method. Neutral detergent fiber (NDF), acid detergent fiber (ADF), and acid detergent lignin (ADL) were determined using an Ankom A2000i fiber analyzer (Ankom A2000i fiber analyzer (Ankom Technology, Macedon, NY, USA)) according to the method described by Van Soest et al. [25]. Cellulose content was calculated as the difference between ADF and ADL, whereas hemicellulose content was calculated as the difference between NDF and ADF. Dry matter loss and changes in the absolute amounts of TP, cellulose, hemicellulose, and ADL were calculated by comparing sample weights before and after incubation. The degradation rate of each component was calculated from its absolute amount as follows: degradation rate (%) = [(initial absolute amount − absolute amount after fermentation)/initial absolute amount] × 100.

For pH determination, 1 g of fresh sample was mixed with 30 mL of deionized water and shaken at 230 rpm for 30 min. The pH of the extract was then measured using a pH meter (Herisau Metrohm, Herisau, Switzerland).

### 2.4. Total RNA Extraction, Quality Assessment, Transcriptome Library Construction, and Sequencing

Approximately 100 mg of each sample stored at −80 °C was thoroughly ground in liquid nitrogen, followed by the addition of 1 mL of TRIzol reagent (Invitrogen, Carlsbad, CA, USA). After complete lysis, the homogenate was incubated at room temperature for 5 min and then centrifuged at 13,000× *g* for 5 min at 4 °C. The supernatant was collected, mixed with 0.2 mL of chloroform, and incubated at room temperature for 5 min. After centrifugation at 13,000× *g* for 15 min at 4 °C, approximately 400 μL of the aqueous phase was transferred to a new tube, mixed with an equal volume of isopropanol, and incubated at room temperature for 10 min. The mixture was then centrifuged at 13,000× *g* for 10 min at 4 °C. The supernatant was discarded, and the RNA pellet was washed with 1 mL of 75% ethanol. After centrifugation at 12,000× *g* for 5 min at 4 °C, the supernatant was removed, and the RNA pellet was air-dried and dissolved in DEPC-treated water.

RNA concentration and purity were assessed using a NanoDrop 2000 spectrophotometer (Thermo Fisher Scientific, Waltham, MA, USA). RNA integrity was evaluated by 1% agarose gel electrophoresis (Biowest, Nuaillé, France), and RNA quality number (RQN) values were determined using an Agilent 2100 Bioanalyzer (Agilent Technologies, Santa Clara, CA, USA). Samples used for library construction met the following criteria: total RNA amount ≥ 1 μg, RNA concentration ≥ 30 ng/μL, RQN > 6.5, OD260/280 = 1.8–2.2, and OD260/230 ≥ 1.5.RNA enrichment, fragmentation, reverse transcription, library construction, and sequencing were performed by Majorbio BioPharm Technology Co., Ltd. (Shanghai, China) according to the manufacturer’s instructions.

Transcriptome libraries were constructed using the Illumina^®^ Stranded mRNA Prep Ligation kit (Illumina, San Diego, CA, USA). Briefly, mRNA was enriched using oligo(dT) magnetic beads and fragmented to approximately 300 bp using fragmentation buffer. First strand cDNA was synthesized using random primers and reverse transcriptase, followed by second strand cDNA synthesis in which dUTP was incorporated in place of dTTP. The double stranded cDNA was subjected to end repair, Atailing, and ligation with Y-shaped adapters. The dUTP-containing second strand was then degraded using UDG enzyme. The resulting products were purified using magnetic beads, size selected, and amplified by PCR to generate the final libraries. Library quality was assessed using a Qubit 4.0 fluorometer (Thermo Fisher Scientific, Waltham, MA, USA), and fragment size distribution was evaluated using an Agilent 2100 Bioanalyzer (Agilent Technologies, Santa Clara, CA, USA). Qualified libraries were pooled and subjected to bridge PCR amplification on a cBot system to generate clusters. Paired end sequencing with a read length of 150 bp was performed on a NovaSeq X Plus platform (Illumina, San Diego, CA, USA).

### 2.5. Metabolite Extraction and UHPLC–MS/MS Analysis

Metabolomic analysis was performed using a UHPLC–Q Exactive HF-X system (Thermo Fisher Scientific, Waltham, MA, USA) equipped with an ACQUITY UPLC HSS T3 column (100 mm × 2.1 mm, 1.8 μm; Waters, Milford, MA, USA). Approximately 100 mg of solid sample was accurately weighed into a 2 mL centrifuge tube, followed by the addition of one 6 mm grinding bead and 800 μL of extraction solvent (methanol:water = 4:1, *v*/*v*) containing four internal standards: L-2-chlorophenylalanine (0.02 mg/mL), palmitoyl-L-carnitine-(N-methyl-d_3_), cholic acid-2,2,4,4-d_4_, and L-phenylalanine-d_5_.

The samples were ground at −10 °C and 50 Hz for 6 min using a Wonbio-96c instrument (Shanghai Wonbio Biotechnology Co., Ltd., Shanghai, China), followed by ultrasonic extraction at 5 °C and 40 kHz for 30 min. The extracts were then cooled at −20 °C for 30 min and centrifuged at 13,000× *g* for 15 min at 4 °C. The supernatant was collected for instrumental analysis. In addition, 100 μL of supernatant from each sample was pooled to prepare a quality control (QC) sample. The mobile phase consisted of solvent A, 95% water and 5% acetonitrile containing 0.1% formic acid, and solvent B, 47.5% acetonitrile, 47.5% isopropanol, and 5% water containing 0.1% formic acid. The gradient elution program was as follows: 0–2 min, 5% B; 2–12 min, 5–95% B; 12–14 min, 95% B; 14–14.1 min, 95–5% B; and 14.1–16 min, 5% B for column equilibration. The flow rate was 0.40 mL/min, the column temperature was maintained at 40 °C, and the injection volume was 3 μL. Mass spectrometric detection was performed using an electrospray ionization (ESI) source in both positive and negative ion modes. The scan range was set to m/z 70–1050. The spray voltage was set to 3500 V in positive ion mode and −3000 V in negative ion mode. The sheath gas and auxiliary gas were set to 50 and 13 arbitrary units, respectively. The ion source temperature was maintained at 450 °C, and collision energy was applied in a 20–40–60 V rolling mode.

### 2.6. Transcriptome Data Processing and Bioinformatic Analysis

Raw sequencing reads were processed using fastp (v0.23.4) to remove adapter sequences, low-quality bases (Q ≤ 20), and reads containing more than 10% ambiguous bases (N). Reads shorter than 20 bp after trimming were discarded. The clean reads were aligned to the *G*. *lucidum* reference genome using HISAT2 (v2.1.0). Gene and transcript expression levels were quantified using RSEM (v1.3.3) and expressed as FPKM/TPM values. Differential expression analysis was performed using DESeq2(v1.42.0). Genes with |log_2_FC| ≥ 1 and an adjusted *p*-value (*p*_adj) < 0.05 were considered differentially expressed genes (DEGs). Gene Ontology (GO) annotation was performed using DIAMOND (v2.0.11), and GO enrichment analysis was conducted using Goatools (v1.4.4) with Fisher’s exact test. GO terms with *p*_fdr ≤ 0.05 were considered significantly enriched. Kyoto Encyclopedia of Genes and Genomes (KEGG) pathway enrichment analysis was performed using KOBAS (v3.0), and pathways with adjusted *p* < 0.05 were considered significantly enriched.

### 2.7. Metabolome Data Processing and Bioinformatic Analysis

Raw LC–MS data were imported into Progenesis QI software v3.0 (Waters, Milford, MA, USA) for peak detection, peak alignment, retention time correction, and peak area normalization. Metabolites were identified by MS/MS spectral matching against the Human Metabolome Database (HMDB; http://www.hmdb.ca/, accessed on 1 May 2026), METLIN (https://metlin.scripps.edu/, accessed on 1 May 2026), and the Majorbio in-house database. The data matrix was preprocessed by removing missing values according to the 80% rule, imputing remaining missing values with the minimum value, and applying total sum normalization. Variables with a relative standard deviation greater than 30% in quality control (QC) samples were removed, followed by log10 transformation. The untargeted metabolomic analysis was based on normalized peak intensities and provided relative rather than absolute quantification. Compound specific calibration curves were not established for all detected metabolites. Principal component analysis (PCA) and orthogonal partial least squares discriminant analysis (OPLS-DA) were performed using the ropls package (v1.6.2) in R. Model stability was assessed using seven-fold cross-validation. Differential metabolites (DMs) were screened based on variable importance in projection (VIP) > 1 in the OPLS-DA model and *p* < 0.05 by Student’s *t*-test. KEGG pathway annotation of DMs was performed using the KEGG database, and pathway enrichment analysis was conducted using Fisher’s exact test in the scipy.stats package in Python v1.0.0. Pathways with *p* < 0.05 were considered significantly enriched.

### 2.8. Statistical Analysis

Data on pH and chemical composition were analyzed using the general linear model (GLM) procedure in SAS 9.4. The statistical model was as follows: Yijk = μ + Ti + Dj + (T × D)ij + εijk, where Yijk represents the observation under temperature i and fermentation duration j, μ is the overall mean, Ti is the fixed effect of temperature, Dj is the fixed effect of fermentation duration, (T × D)ij is the interaction effect between temperature and fermentation duration, and εijk is the random error. Multiple comparisons among treatments were performed using Tukey’s test at α = 0.05. Data on pH and cell wall composition included three biological replicates.

Omics data were analyzed using the Majorbio Cloud Platform (https://cloud.majorbio.com, accessed on 1 May 2026). Integrated transcriptomic and metabolomic analyses were conducted by constructing correlation networks based on Spearman correlation coefficients. Gene–metabolite association mapping was further performed by integrating differentially expressed genes (DEGs), differential metabolites (DMs), and their coenriched pathways in the KEGG database.

## 3. Results

### 3.1. Changes in pH and Chemical Composition of Wheat Straw Fermented by G. lucidum at Different Temperatures

This study evaluated changes in pH and the contents of true protein, cellulose, hemicellulose, and lignin in wheat straw after solid-state fermentation by *G. lucidum* at 20 °C, 27 °C, and 34 °C for 0, 2, 4, and 6 weeks (Table 1). Incubation temperature and fermentation duration significantly affected the pH of the fermentation system and the absolute amounts of major structural components in wheat straw (*p* < 0.05). Across all treatments, pH decreased continuously with increasing fermentation duration, from an initial value of 6.12 at week 0 to 4.38–4.58 at the end of fermentation. Specifically, at 20 °C, pH decreased gradually and reached 4.58 at week 6. At 27 °C, pH decreased rapidly to 4.64 by week 2, reached the lowest value of 4.38 at week 4, and then slightly increased to 4.46 at week 6. At 34 °C, pH decreased to 4.58 by week 2 and subsequently remained relatively stable, with values of 4.54 and 4.55 at weeks 4 and 6, respectively.

Analysis of wheat straw composition showed that true protein concentration initially increased from 28.1 g kg^−1^ DM in the control to 35.8 g kg^−1^ DM at 20 °C at week 4 and to 34.5 g kg^−1^ DM at 27 °C during week 2. However, the absolute amount of true protein decreased over time, particularly at 34 °C, where it decreased from 1.87 g to 1.43 g after 6 weeks, and at 27 °C, where it decreased to 1.57 g after 6 weeks. Except for the significantly lower true protein content observed after 6 weeks of fermentation at 34 °C compared with the control (*p* < 0.05), no significant differences in true protein content were observed between the other treatments and the control (*p* > 0.05). The degradation rates of cellulose, hemicellulose, and lignin increased with fermentation duration, showing a clear time-dependent pattern. Overall, the degradation of cellulose, hemicellulose, and lignin was greater at 27 °C and 34 °C than at 20 °C. After 6 weeks of fermentation, hemicellulose content decreased from the initial value of 335.8 g kg^−1^ to 252.6, 194.9, and 208.7 g kg^−1^ at 20 °C, 27 °C, and 34 °C, respectively, corresponding to absolute degradation rates of 33.20%, 59.78%, and 59.87%. Lignin content decreased from the initial value of 58.4 g kg^−1^ to 48.6, 30.5, and 33.4 g kg^−1^ at 20 °C, 27 °C, and 34 °C, respectively, with absolute degradation rates of 26.97%, 63.61%, and 63.61%. Although fermentation temperature and duration did not significantly affect the relative cellulose content (*p* > 0.05), the absolute cellulose degradation rates after 6 weeks increased to 21.59%, 39.65%, and 46.18% at 20 °C, 27 °C, and 34 °C, respectively. Dry matter loss showed a similar trend to cellulose degradation, reaching 11.01%, 30.73%, and 35.20% at 20 °C, 27 °C, and 34 °C, respectively.

### 3.2. Transcriptomic Responses of G. lucidum During Wheat Straw Fermentation at Different Temperatures

**Figure 1:**
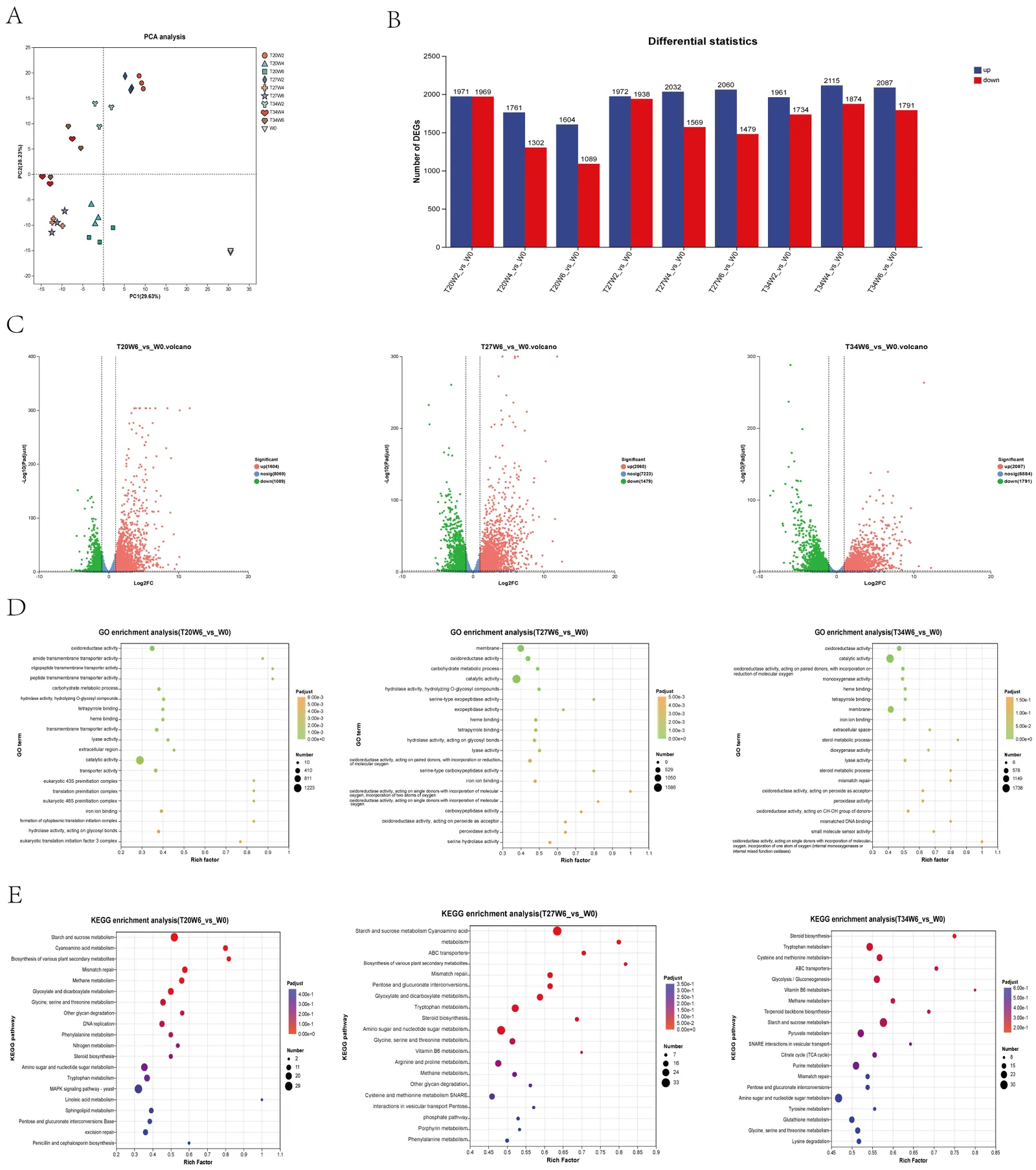
Transcriptomic responses of *Ganoderma lucidum* during wheat straw fermentation at different temperatures (T) and duration (W). (**A**) Principal component analysis (PCA) score plot. (**B**) Numbers of up-regulated and down-regulated differentially expressed genes (DEGs) at different fermentation time points compared with W0. (**C**) Volcano plots of DEGs. (**D**) GO enrichment analysis of DEGs. (**E**) KEGG pathway enrichment analysis of DEGs.

To characterize the transcriptomic responses of *G. lucidum* during solid-state fermentation of wheat straw at different temperatures, RNA-seq was performed on samples collected after 0, 2, 4, and 6 weeks of fermentation at 20 °C, 27 °C, and 34 °C. Principal component analysis (PCA) showed that the first two principal components explained 57.86% of the total variance (Figure 1A). Biological replicates clustered closely, indicating good reproducibility and data stability. The samples exhibited clear separation according to fermentation time and temperature. Within each temperature treatment, the largest transcriptomic shift occurred between week 0 and week 2, whereas samples from weeks 4 and 6 were relatively close to each other. Using week 0 as the control, differentially expressed genes (DEGs) were identified using |log2FC| ≥ 1 and *p*_adj < 0.05 as screening criteria. At 20 °C, the total number of DEGs decreased with fermentation duration, with 3940, 3063, and 2693 DEGs detected at weeks 2, 4, and 6, respectively (Figure 1B). A similar trend was observed at 27 °C, with 3910, 3601, and 3539 DEGs detected at weeks 2, 4, and 6, respectively. In contrast, the number of DEGs at 34 °C increased from 3695 at week 2 to 3989 at week 4 and then slightly decreased to 3878 at week 6. At week 6, the total number of DEGs increased with temperature in the order 34 °C > 27 °C > 20 °C, suggesting that higher incubation temperatures induced a broader transcriptional response.

After 6 weeks of fermentation, the number of up-regulated genes exceeded the number of down-regulated genes under all three temperature conditions (Figure 1C). Specifically, 1604 genes were up-regulated and 1089 were down-regulated at 20 °C, 2060 genes were up-regulated and 1479 were down-regulated at 27 °C, and 2087 genes were up-regulated and 1791 were down-regulated at 34 °C. The number of up-regulated genes increased with temperature. Analysis of lignocellulose degradation-related genes showed that *G. lucidum* activated both lignin-modifying and carbohydrate-degrading enzyme genes under all temperature conditions. For lignin degradation, laccase-related genes (*LAC1* and *EFM2*) were up-regulated after 6 weeks under all three temperatures, whereas *FER1* was co-up-regulated at 20 °C and 27 °C. For carbohydrate-active enzymes (CAZymes), multiple genes associated with cellulose, hemicellulose, and pectin degradation were commonly up-regulated under all three temperatures, including endocellulase (*XGEA*), endoglucanases (*GUN2* and *GUN3*), endo-β-1,4-glucanase (*xynA*), β-glucosidase 2 (*bglX*), cellobiohydrolase I (*GUX1*), pectin lyase-like protein (*RGXA*), endoglucanase V-like protein (*GUN5*), β-xylosidase (*BXLB*), β-mannosidase B (*manB*), and several glycoside hydrolase genes. Notably, xylitol dehydrogenase (*XYL2*) was specifically up-regulated at 27 °C, whereas barwin-like endoglucanase (*PAPI*) was specifically up-regulated at 34 °C; however, *GUN3* and *FER1* were not up-regulated at 34 °C.

### 3.3. GO and KEGG Pathway Enrichment Analysis

To further clarify the functional characteristics of the transcriptomic response during wheat straw degradation by *G. lucidum*, GO and KEGG enrichment analyses were performed for DEGs at week 6 relative to week 0 under three tested temperatures (Figure 1D,E; Table S1). In the molecular function category, DEGs in all three treatments were significantly enriched in oxidoreductase activity, heme/tetrapyrrole binding, iron ion binding, peroxidase activity, monooxygenase/dioxygenase activity, hydrolase activity, and lyase activity. In the biological process category, all three treatments were enriched in carbohydrate and polysaccharide metabolic processes, oxidative stress response, and formate metabolic processes. At 20 °C and 27 °C for six weeks of fermentation, DEGs were enriched in processes associated with polysaccharide, cell wall, translation initiation, formate metabolism, and catechol-containing compound metabolism, including β-glucan, glucan, and cellulose metabolic processes, polysaccharide catabolism, formate catabolism/metabolism, and catechol-containing compound metabolism. In addition, at 27 °C for six weeks, DEGs showed specific enrichment in sterol/steroid biosynthesis, methionine biosynthesis, chitin catabolism, and sulfur-containing amino acid metabolism. In contrast, at 34 °C for six weeks, DEGs were more prominently enriched in mismatch repair, small molecule metabolism, terpenoid-related processes, pyruvate metabolism, and purine nucleoside diphosphate-related processes.

KEGG pathway enrichment analysis showed that, after 6 weeks of fermentation, the top 20 enriched pathways of DEGs under all three temperatures were mainly associated with carbohydrate metabolism, amino acid metabolism, and biosynthesis of secondary metabolites. However, the enrichment patterns differed among temperatures. At 20 °C, DEGs were significantly enriched in amino acid biosynthesis and metabolism, including cyanoamino acid, tryptophan, phenylalanine, glycine, serine, and threonine metabolism, as well as nitrogen metabolism. Pathways related to carbohydrate degradation and metabolism, glyoxylate and dicarboxylate metabolism, steroid biosynthesis, and biosynthesis of various plant secondary metabolites were also enriched. At 27 °C, the transcriptomic profile shifted toward activation of complex carbohydrate degradation, energy metabolism, and transport-related pathways, including the pentose phosphate pathway, pentose and glucuronate interconversions, glyoxylate and dicarboxylate metabolism, and ABC transporters. Several amino acid metabolism pathways, vitamin B6 metabolism, steroid biosynthesis, biosynthesis of various plant secondary metabolites, other glycan degradation, and amino sugar and nucleotide sugar metabolism were also enriched. At 34 °C, carbohydrate degradation and metabolism remained active, while glycolysis/gluconeogenesis, pyruvate metabolism, the tricarboxylic acid (TCA) cycle, and purine metabolism were strongly enriched. Terpenoid backbone biosynthesis, steroid biosynthesis, tryptophan metabolism, cysteine and methionine metabolism, and vitamin B6 metabolism were also enriched.

### 3.4. Effects of Temperature on the Metabolomic Profiles of Wheat Straw During Solid-State Fermentation by G. lucidum

**Figure 2:**
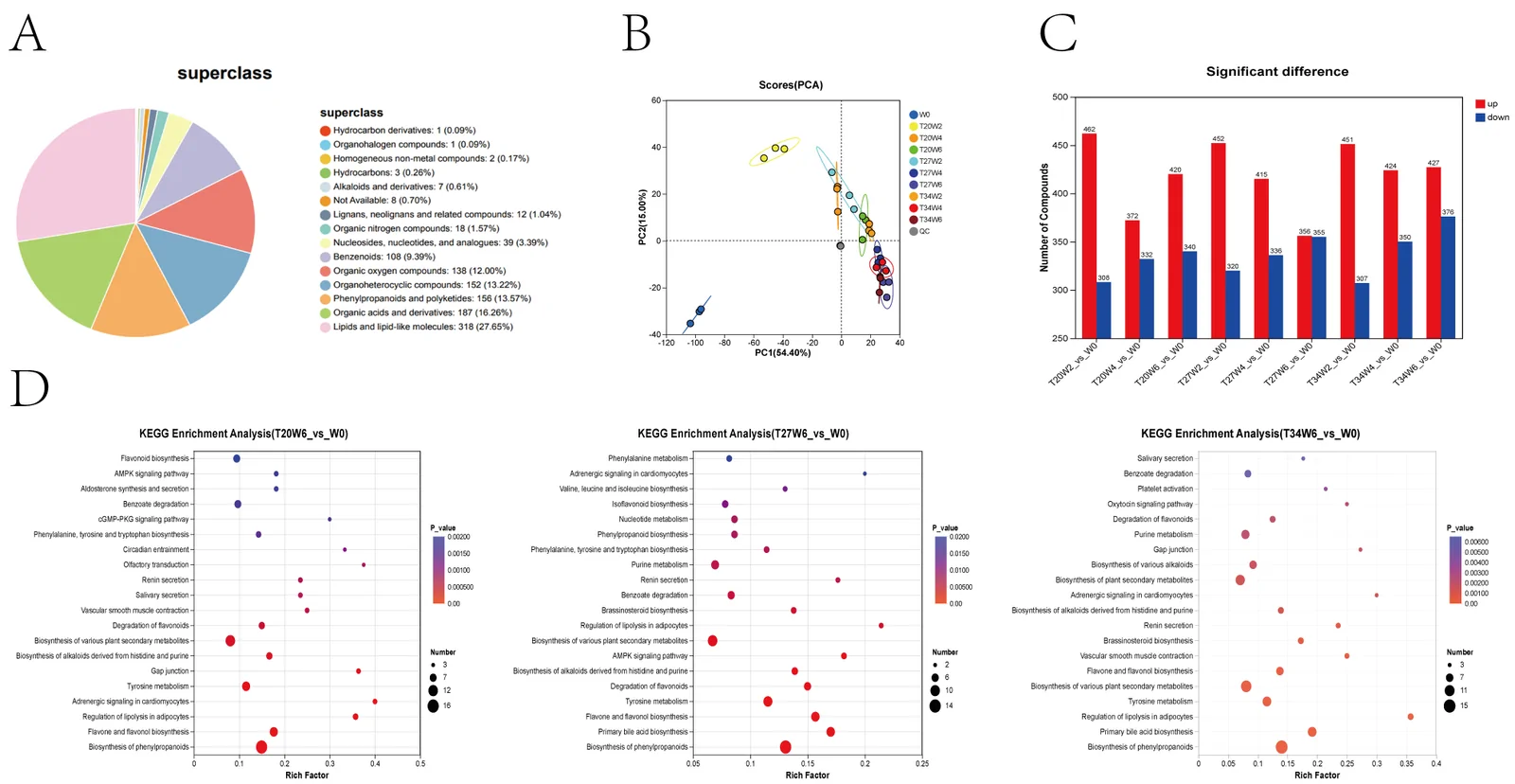
Metabolomic profiles of wheat straw fermented by *Ganoderma lucidum* at different temperatures (T) and duration (W). (**A**) Superclass classification of identified metabolites based on the Human Metabolome Database. (**B**) Principal component analysis (PCA) score plot of metabolomic profiles from different treatment groups. Different-colored dots represent samples from different treatment groups; ellipses indicate the 95% confidence intervals for each group; and lines connect the sample points to the group centers. (**C**) Numbers of up-regulated and down-regulated differential metabolites in each treatment compared with W0. (**D**) KEGG pathway enrichment analysis of differential metabolites.

To investigate metabolic changes during wheat straw fermentation by *G. lucidum* at different temperatures, untargeted LC–MS/MS-based metabolomic analysis was performed on unfermented wheat straw and straw fermented for 2, 4, and 6 weeks at 20 °C, 27 °C, and 34 °C. A total of 4437 and 6922 ion features were detected in positive and negative ion modes, respectively. Database and MS/MS spectral matching resulted in the annotation of 1579 metabolites, including 838 in positive ion mode and 741 in negative ion mode. Because individual metabolites may generate multiple adducts, isotopes, or fragment signals, the number of detected ion features does not correspond directly to the number of unique metabolites. Based on HMDB classification (Figure 2A), the identified metabolites were assigned to lipids and lipid-like molecules (318), organic acids and derivatives (187), phenylpropanoids and polyketides (156), organoheterocyclic compounds (152), organic oxygen compounds (138), benzenoids (108), nucleosides, nucleotides, and analogs (39), organic nitrogen compounds (18), lignans, neolignans, and related compounds (12), alkaloids and derivatives (7), hydrocarbons (3), homogeneous nonmetal compounds (2), hydrocarbon derivatives (1), organohalogen compounds (1), and unclassified compounds (8).

PCA showed that the first two principal components explained 69.4% of the total variance (PC1, 54.4%; PC2, 15.0%; Figure 2B). Quality control samples clustered tightly, indicating good reproducibility and stability of the metabolomic data. Samples from week 0 formed a distinct cluster on the left side of PC1 and were clearly separated from all fermented samples. Under each temperature condition, samples from different fermentation times showed a continuous metabolic trajectory along PC2, with the largest metabolic shift occurring between weeks 0 and 2. The samples at week 2 under 20 °C were also clearly separated from the other treatments. Notably, partial metabolic convergence was observed among different temperature–time combinations. Samples at week 4 under 20 °C were close to samples at week 2 under 27 °C. Samples at week 6 under 20 °C were close to samples at week 2 under 34 °C. Samples at week 4 and week 6 under 27 °C were close to samples at week 4 and week 6 under 34 °C, respectively. These patterns suggest that prolonged low-temperature fermentation can produce metabolomic profiles similar to those generated by shorter high-temperature fermentation. However, this convergence appeared to weaken when the temperature reached 34 °C for a prolonged fermentation.

The metabolite abundance increased during the early fermentation stage and then gradually decreased. Lipids and lipid-like molecules and hydrocarbons showed particularly clear changes; compared with 20 °C, these compound classes tended to decrease with fermentation duration at 27 °C and 34 °C. Organic acids and derivatives were more abundant in fermented samples than in week 0, but no consistent trend was observed across temperatures and time points. This pattern was not fully consistent with the pH dynamics. Nucleosides, nucleotides, and analogs; lignans, neolignans, and related compounds; and phenylpropanoids and polyketides decreased relative to week 0. Nucleosides, nucleotides, and analogues gradually decreased with fermentation duration. Lignans, neolignans, and related compounds and phenylpropanoids and polyketides decreased over time at 20 °C, whereas their trends were less evident at 27 °C and 34 °C. Benzenoids increased gradually at 20 °C but showed a weaker increase at 27 °C and 34 °C. In addition, amino acids/peptides, carbohydrates, triterpenoids, sesquiterpenoids, carbonyl compounds, and fatty acids increased relative to week 0, whereas flavonoid glycosides decreased.

### 3.5. Effects of Temperature on Differential Metabolites and Metabolic Pathways During Wheat Straw Fermentation by G. Lucidum

To elucidate the differential metabolites and their associated metabolic pathways during wheat straw fermentation by *G*. *lucidum* under different temperatures, metabolomic profiling and KEGG pathway enrichment analysis were performed on samples fermented for 6 weeks at 20 °C, 27 °C, and 34 °C in comparison with the unfermented control (week 0). Differential metabolites (DMs) were screened using the criteria of variable importance in projection (VIP) > 1 and *p* < 0.05. Volcano plot analysis based on orthogonal partial least squares discriminant analysis (OPLS-DA) (Supplementary Figure S1) revealed that, among the top 30 metabolites ranked by VIP values, the common metabolites shared across the three comparisons were predominantly flavonoids, organic acids, nucleotides, and coumarins.

At 20 °C after six weeks of fermentation compared with week 0, 760 DMs were identified, including 420 up-regulated and 340 down-regulated metabolites. Seven metabolites were significantly up-regulated, including thioctic acid, nonanoic acid, monoglyceride citrate, 4-hydroxy-benzenepropanedioate, cystathionine, 3-hydroxycoumarin, and tuliposide A. At 27 °C after six weeks, 711 DMs were identified, including 356 up-regulated and 355 down-regulated metabolites. The top 30 VIP-ranked up-regulated metabolites included monoglyceride citrate, thioctic acid, 2-O-caffeoyltartronic acid, 2-hydroxy-5-(trifluoromethoxy)benzoic acid, 3-hydroxycoumarin, methylnorlichexanthone, and 4-nitrophenyl beta-D-glucopyranosiduronic acid. At 34 °C after six weeks, 803 DMs were identified, including 427 up-regulated and 376 down-regulated metabolites. The top 30 VIP-ranked up-regulated metabolites included monoglyceride citrate, thioctic acid, 2-O-caffeoyltartronic acid, 2-hydroxy-5-(trifluoromethoxy)benzoic acid, 3-hydroxycoumarin, methylnorlichexanthone, and 4-nitrophenyl beta-D-glucopyranosiduronic acid. Thioctic acid, monoglyceride citrate, and 3-hydroxycoumarin were commonly up-regulated across all three temperature treatments.

KEGG enrichment analysis (Figure 2D) showed that DMs in all three comparisons were mainly enriched in the biosynthesis of phenylpropanoids, flavone and flavonol biosynthesis and degradation, tyrosine metabolism, biosynthesis of various plant secondary metabolites, and benzoate degradation. At 20 °C after six weeks of fermentation compared with week 0, specific enrichment in isoflavonoid biosynthesis, valine, leucine and isoleucine biosynthesis, and phenylalanine metabolism was observed.

### 3.6. Integrated Transcriptomic and Metabolomic Analysis Under Different Temperature Conditions

**Figure 3:**
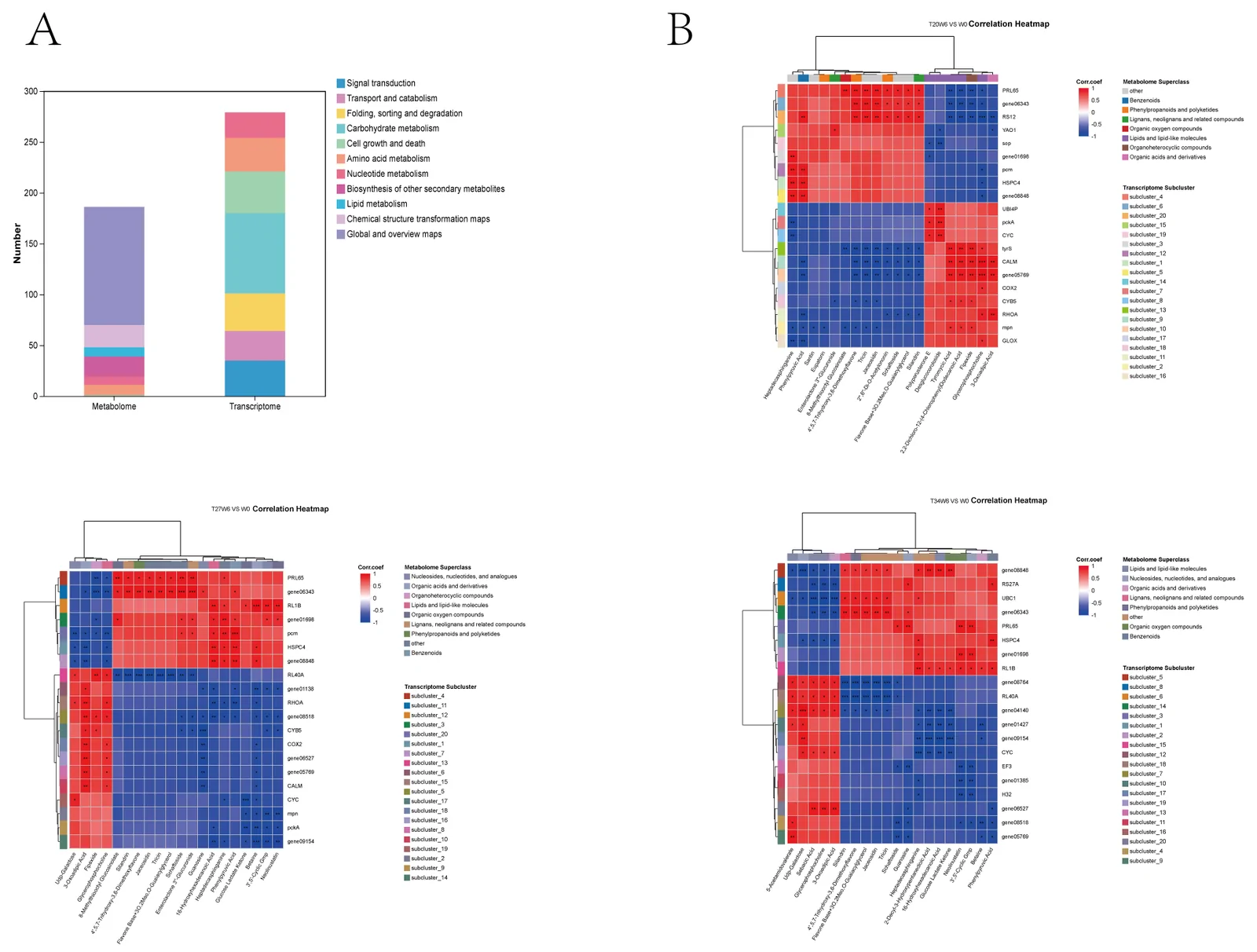
(**A**) KEGG pathway level two classification derived from the integrated analysis of transcriptome and metabolome. (**B**) Clustered heatmap of correlations between transcriptomic and metabolomic datasets. The following symbols indicate statistical significance using Spearman’s rank correlation test, * *p* < 0.05, ** *p* < 0.01, and *** *p* < 0.001.

In this study, correlation analyses between DEGs and DMs were performed under different temperature treatments. KEGG pathway level two classification of the integrated results (Figure 3A) showed that pathway enrichment was generally more significant at the metabolomic level than at the transcriptomic level. Under all three temperature conditions, DEGs and DMs were co-enriched in pathways related to signal transduction, transport and catabolism, cell growth and death, amino acid metabolism, carbohydrate metabolism, biosynthesis of other secondary metabolites, and chemical structure transformation, indicating that these pathways are essential for maintaining basic cellular functions of the white-rot fungus during solid-state fermentation of wheat straw. Notably, the metabolism and utilization of carbon sources from straw, as well as the production of secondary metabolites, occurred continuously throughout the fermentation process. At higher fermentation temperatures (27 °C and 34 °C), DEGs and DMs were additionally significantly enriched in pathways related to folding, sorting and degradation, lipid metabolism, and nucleotide metabolism. Furthermore, 90, 37, and 59 significantly enriched pathways (*p* < 0.05) were identified at 20 °C, 27 °C, and 34 °C, respectively.

Spearman correlation analysis was performed to calculate the correlation coefficients and *p*-values between metabolites and DEGs. The top 20 gene–metabolite associations were visualized using a clustering heatmap (Figure 3B). Across the three temperature conditions, the gene–metabolite correlation networks exhibited temperature-dependent features, along with conserved antagonistic relationships. A common feature across all three temperature treatments was the persistent antagonism between phenylpropanoids/polyketides/lignans and lipids/lipid-like molecules/organic acids and their derivatives. Genes such as *PRL65* (encoding a predicted sphingomyelin phosphodiesterase) and uncharacterized candidate nodes *gene06343* and *gene08848* showed significant positive correlations with 4’,5,7-trihydroxy-3,6-dimethoxyflavone and silandrin, but negative correlattions with glycerophosphocholine and 3-oxoadipic acid. In contrast, genes such as *CALM* (encoding calmodulin) and *gene05769* displayed opposite association patterns, positively correlated with glycerophosphocholine and 3-oxoadipic acid, but negatively correlated with the aforementioned flavonoids and lignans. Additionally, glycerophosphocholine consistently emerged as a representative negatively correlated metabolite in lipid metabolism across multiple temperatures, while 3-oxoadipic acid served as a central negatively correlated metabolite within organic acids.

Temperature significantly reshaped key gene–metabolite interaction nodes, specifically through shifts in the types of metabolites and genes associated positively or negatively under different temperature conditions. At 20 °C, *RS12* (encoding ribosomal protein) showed a significant negative correlation with 3-oxoadipic acid (*p* < 0.01), whereas *pcm* (encoding protein-L-isoleucine O-methyltransferase), *HSPC4* (encoding small heat shock protein), and *gene08848* were highly positively correlated with phenylpyruvic acid, a benzenoid compound. Additionally, *UBI4P* (encoding ubiquitin/polyubiquitin-like proteins), *pckA* (encoding phosphoenolpyruvate carboxykinase), and *CYC* (encoding cytochrome c) showed positive correlations with lipid metabolites polyporusterone E and desglucocoroloside. At 27 °C, *RL1B* (encoding predicted ribosomal large subunit protein L10Ae family member), *HSPC4*, and *gene08848* showed positive correlations with the lipid 16-hydroxyhexadecanoic acid, whereas *RHOA* (encoding GTPase), gene08518, *COX2* (encoding cytochrome P450 monooxygenase), *gene06527*, *gene05769*, and *CALM* were positively correlated with 3-oxoadipic acid and glycerophosphocholine but negatively correlated with betaine. Meanwhile, *mpn* (encoding manganese peroxidase), *pckA*, and *gene09154* were negatively correlated with betaine and positively correlated with 3-oxoadipic acid. At 34 °C, *gene08848*, *RS27A* (encoding ubiquitin-40S ribosomal protein S27a fusion protein), and *gene06343* showed negative correlations with 5-acetamidovalerate, sebacic acid, glycerophosphocholine, and 3-oxoadipic acid, but positive correlations with betaine; meanwhile, *gene08764*, *RL40A* (encoding ubiquitin-60S ribosomal protein L40 fusion protein), and *gene04140* were positively correlated with sebacic acid, glycerophosphocholine, and other lipids, as well as with silandrin and 4’,5,7-trihydroxy-3,6-dimethoxyflavone.

## 4. Discussion

### 4.1. Temperature-Dependent Regulation of the Degradation Phenotype During Wheat Straw Fermentation by G. lucidum

During solid-state fermentation of wheat straw by *G. lucidum*, a pronounced decrease in pH was consistent with previous reports on other white-rot fungi in both liquid and solid cultures [26,27]. This acidification is likely driven by the accumulation of organic acids, such as oxalic, citric, gluconic, and malic acids, which are characteristic metabolites of some selective white-rot fungi [28]. This resulting pH reduction in the vicinity of fungal hyphae serves multiple ecological functions, particularly by creating a suitable acidic environment for the activity of secreted hydrolytic and oxidative enzymes [29]. A previous study reported that all 15 laccases identified in *G. lucidum* exhibited maximum activity at either pH 3 or pH 7 [30]. The most substantial decrease in pH occurred within the first four weeks of fermentation, as also reported by Mao et al. [31]. This might indicate that the majority of organic acid production takes place during this period. Notably, pH was affected by the incubation temperature, with greater pH decreases observed at higher temperatures of 27 °C and 34 °C compared with 20 °C. This contrasts with a previous study on *C. subvermispora*, which showed that incubation at 24 °C reduced the pH of rice straw more than incubation at 30 °C, specifically from 4.9 to 4.0 and 4.4, respectively [21]. This discrepancy suggests that different white-rot fungi may exhibit distinct temperature responses of their acid-producing metabolic pathways to temperature variation, a notion supported by a study showing that fungal growth is influenced by temperature [21]. Furthermore, the slight increase or stabilization of pH during the later stages of fermentation at 27 °C and 34 °C may be attributed to the release of buffering substances resulting from lignin depolymerization.

During the initial screening phase of this study, we also tested the fermentation efficiency of *Ceriporiopsis subvermispora* and *Lentinula edodes* on wheat straw under identical temperature conditions. However, *C. subvermispora* exhibited a long growth cycle and unstable lignocellulose degradation activity at higher temperatures, while *L. edodes* failed to grow properly at 34 °C, making it unsuitable for systematic studies covering the full temperature range of 20–34 °C. Therefore, we ultimately selected the *G. lucidum* G5 strain, despite its lower cellulose selectivity compared to certain highly selective white-rot fungi. Regarding the rationale for including the 34 °C treatment, although lignin and hemicellulose degradation rates were similar at both 34 °C and 27 °C, we specifically chose this temperature based on the following considerations. First, although 34 °C is slightly above the optimal growth temperature range of *G. lucidum* (25–30 °C), it remains within the organism’s survival and metabolic activity range, allowing us to capture distinct moderate heat stress responses at transcriptional and metabolic levels compared to 27 °C. Second, chemical composition data revealed that the cellulose degradation rate at 34 °C (46.18%) was significantly higher than that at 27 °C (39.65%), indicating differential temperature sensitivity among various cell wall components—a contrast that provides crucial insights for the multi-omics analysis of temperature-dependent metabolic reprogramming.

The absolute amount of true protein decreased most markedly after six weeks of fermentation at 34 °C, although changes in true protein concentration were less consistent among treatments. As defined by Sniffen et al. [32], the crude protein fraction consists of non-protein nitrogen, true protein, and unavailable nitrogen. Fungi degrade carbohydrates to CO_2_ and H_2_O during incubation, resulting in a loss of organic matter but not of nitrogen at different temperatures [21,31]. The observed reduction in true protein necessarily implies a corresponding increase in non-protein nitrogen. Under high-temperature conditions, this shift may be attributed to heat stress-induced ammonia volatilization, changes in nitrogen utilization pathways [33], or protein degradation under the 34 °C condition. Moreover, fermentation at 27 and 34 °C significantly accelerated the breakdown of lignin and structural carbohydrates compared to 20 °C, suggesting that 27–34 °C may be closer to the optimal temperature range for the ligninolytic enzyme system of *G. lucidum*. This inference is supported by the finding that the highest laccase activity was observed at 31 °C when compared with that at 25, 28 and 34 °C [34]. Notably, cellulose degradation increased steadily with temperature and peaked at 34 °C, whereas hemicellulose and lignin degradation showed no further increase beyond 27 °C. A similar temperature-dependent variation in cellulose degradation has been reported for other white-rot fungi. Hai et al. [21] found that after six weeks of incubation on rice straw, cellulose degradation by *L. edodes* was 16.7% at 30 °C compared to 7.2% at 24 °C, whereas *C. subvermispora* showed no significant difference between the two temperatures. Interestingly, despite these differences in cellulose degradation, in vitro gas production did not vary significantly across temperatures for either fungus in that study. This may be because changes in ADL content have a greater influence on changes in in vitro gas production than do changes in cellulose degradation [35].

In this study, cellulose degradation reached 39.65% at 27 °C and 46.18% at 34 °C, while lignin degradation was 63% under both incubation temperatures. Considering the comparable lignin and hemicellulose degradation at 27 °C and 34 °C, but significantly higher cellulose loss at 34 °C (46.18% vs. 39.65%), 27 °C is recommended as the optimal incubation temperature for pretreating wheat straw with this *G. lucidum* strain, as it achieves the best balance between delignification efficiency and preservation of fermentable carbohydrates. These cellulose degradation rates are substantially higher than those reported for other white-rot fungi. After seven weeks of fermentation on wheat straw, *C. subvermispora* removed 52.3% of lignin but only 6% of cellulose [36], while *L. edodes* degraded 68% of lignin and 12.3% of cellulose after eight weeks of fermentation [35]. However, in a subsequent study by Nayan et al. [37]. using the same fungal strains, the maximum lignin degradation reached 93.8% with *C. subvermispora* and 80% with *L. edodes*. This variation was primarily attributed to differences in wheat straw cultivars. Specifically, only one of the five cultivars exhibited such high lignin degradation, whereas the other four showed much lower values, ranging from 52% to 62% for *C. subvermispora* and from 45% to 59% for *L. edodes*. Similarly, Van Kuijk et al. [2] reported that a *G. lucidum* strain degraded 41.3% of lignin and 8.3% of cellulose after 12 weeks of fermentation on wheat straw. Such a high level of cellulose degradation observed in the present study would normally limits the utility of the fungal-treated straw as animal feed, because cellulose is considered the key source of fermentable energy in lignocellulosic biomass. However, the relationship between cellulose degradation and feed quality is not straightforward. It is noteworthy that higher efficiency in lignin degradation is positively linked to higher in vitro gas production [2], yet the strain used in that study also degraded a significant amount of cellulose compared to untreated straw. In another study, a 15-day solid-state fermentation of wheat straw with *Ganoderma* sp. rckk02 reduced cellulose content, yet this was accompanied by improved gas production, organic matter digestibility, and dry matter intake in goats [15]. These findings suggest that higher cellulose degradation in this study may not necessarily impair the feed quality of fungal-treated biomass. In addition, fermentation of wheat straw with *G. lucidum* can lead to the accumulation of valuable bioactive metabolites [10,11], which may offset the potential limitation associated with cellulose degradation and even serve as a positive feature for ruminant feed. The highly selective degradation achieved by *G. lucidum* is closely associated with its unique enzyme system composition [38]. The genome of *G. lucidum* contains more cellulase genes than that of hemicellulase- and lignin-modifying enzymes, particularly during the development of fruiting bodies [13].

The association between acidification and lignin degradation is consistent with the involvement of acidic metabolites and oxidative reactions in fungal ligninolytic systems, although the present data do not establish a direct causal relationship. Previous studies have shown that oxalic acid can chelate Mn^2+^ and promote the formation of Mn^3+^, thereby driving MnP-catalyzed oxidative cleavage of lignin side chains [39]. These mechanisms are consistent with the enrichment of oxidoreductase activity and iron ion transport observed in the transcriptomic data of this study.

### 4.2. Temperature-Dependent Transcriptomic Changes During Wheat Straw Fermentation by G. lucidum

The transcriptomic response of *G. lucidum* during solid-state fermentation of wheat straw was strongly temperature-dependent. Gene expression shifted most dramatically in the first two weeks, indicating that the early stage is the primary period for transcriptional adjustment. After six weeks, higher temperatures corresponded to more differentially expressed genes (DEGs), and a consistently higher proportion of up-regulated ones, aligning with the greater substrate conversion observed in Table 1. Across all temperatures, genes encoding laccases, cellulases and hemicellulases were up-regulated, confirming that *G. lucidum* activates its core lignocellulolytic enzyme system, while temperature-specific shifts in accessory gene expression revealed distinct adaptive strategies. Notably, the ferric reductase gene (FER1) was coinduced at 20 °C and 27 °C, whereas the barwin-like endoglucanase gene (PAPI) was exclusively up-regulated at 34 °C, where GUN3 and FER1 showed no significant expression. This directly explains the chemical composition results, which show that cellulose degradation peaked at 34 °C, while hemicellulose and lignin degradation occurred mainly at or below 27 °C (Table 1). Specifically, at 34 °C, *G. lucidum* appears to accelerate cellulose hydrolysis via specific endoglucanase, while suppression of certain genes limits hemicellulose and lignin breakdown, leading to their lesser removal under those conditions. In line with a previous study, fungi can partially tailor their molecular responses to different carbon sources, with DEGs encoding polysaccharide-degrading enzymes, transporters, proteases, and monooxygenases [20]. The temperature-dependent variation in degradative enzyme gene composition likely reflects differences in substrate accessibility, redox potential, and carbon allocation strategies [16,20,40,41].

GO and KEGG enrichment analyses further revealed temperature-dependent functional differentiation in *G. lucidum* during straw degradation. The present study shows that oxidoreductase activity, carbohydrate metabolism, and oxidative stress pathways represent core biological processes underlying straw degradation at all tested temperatures, and these processes are the major transcriptional features of fungal straw degradation. At 20 °C, *G. lucidum* enhancing amino acid biosynthesis and nitrogen metabolism. At 27 °C, it prioritized NADPH generation, substrate transport, and precursor supply for secondary metabolism. At 34 °C, it increased energy production and terpenoid or steroid biosynthesis. Specifically, at 27 °C, *G. lucidum* up-regulated sterol or steroid biosynthesis, methionine metabolism, and chitin catabolism. Sterol biosynthesis may help maintain membrane fluidity under temperature stress [42,43]. Methionine metabolism and the transsulfuration pathway provide precursors for glutathione synthesis, enhancing antioxidant defense [44]. In contrast, at 34 °C, it up-regulated genes involved in energy metabolism to cope with increased maintenance energy demands, thereby exhibiting an adaptive transcriptional shift from amino acid biosynthesis toward energy metabolism and DNA repair. Given that 34 °C exceeds the optimal growth range from 25 to 30 °C for *G. lucidum* [45], it might induce a possible heat stress response. It has been reported that when *G. lucidum* was exposed to heat stress, ATP, an energy indicator, increased significantly compared to that in untreated strains [46]. This supports the enrichment of energy production pathways. Similarly, high temperature impairs glycolysis and the TCA metabolism in *L. edodes*, leading to carbon starvation and energy deficiency [18]. Furthermore, terpenoid-related processes are among the prominently enriched processes at 34 °C, which is consistent with the report that heat stress (42 °C) often up-regulates key mevalonate pathways promoting triterpenoid biosynthesis, while cold stress (14 °C) decreases key mevalonate gene expression and triterpenoid content [47].

### 4.3. Temperature-Dependent Metabolomic Changes During Wheat Straw Fermentation by G. lucidum

Untargeted metabolomic analysis resulted in the annotation of 1579 metabolites, including lipids, organic acids, phenylpropanoids, lignans, nucleotides, and terpenoid-related compounds, indicating extensive metabolic remodeling during wheat straw fermentation by *G. lucidum*. Prolonged fermentation at lower temperature produced a metabolic state resembling that from shorter higher temperature fermentation. For example, the metabolomic profile at 26 °C for 6 weeks partially converged with that of 34 °C for 2 weeks. However, extending fermentation at 34 °C weakened this convergence, suggesting a nonlinear interaction between temperature and fermentation duration, likely due to metabolic homeostasis imbalance induced by heat stress [18]. The summed relative signal intensity of the annotated metabolites increased during the early fermentation stage and subsequently declined, consistent with rapid fungal colonization and substrate conversion [31,35,36]. Notably, organic acid-related signals did not fully follow the pH trajectory, suggesting that acidification was associated with changes in acid composition rather than simply their summed relative abundance. Zhou et al. [13] reported that citric acid increased at high temperature while other organic acids decreased.

Among the top 30 differential metabolites ranked by VIP values, phenylpropanoids, organic acids, and nucleotide-related compounds were shared across the three fermentation groups. These shared metabolites include thioctic acid, monoglyceride citrate, and 3-hydroxycoumarin, all of which were significantly up-regulated, indicating that these three metabolites represent core temperature independent products of *G. lucidum* in response to the straw fermentation environment. Thioctic acid is a potent antioxidant that participates in mitochondrial energy metabolism and oxidative stress defense [48]. Similarly, 3-Hydroxycoumarin, a coumarin class compound with antimicrobial and antioxidant activities [49], may help inhibit the growth of competing microorganisms in the fermentation system through its accumulation, thereby facilitating *G. lucidum* colonization and substrate degradation. Pathway enrichment analysis revealed that the phenylpropanoid biosynthesis pathway, which serves as the core network for the synthesis of phenolic compounds such as lignin, flavonoids, and coumarins in plants and fungi [50,51], was significantly enriched across all three treatments. Its activation at all temperatures indicates that phenylpropanoid metabolites play a critical role in both lignocellulose degradation and antioxidant defense in *G. lucidum*. Consistent with this, the common up-regulation of flavone and flavonol compounds across the three temperatures further confirmed the central role of these compounds in the fermentation system.

Different temperature treatments induced distinct metabolic strategies in *G.* *lucidum*. At 20 °C, cystathionine accumulated. Cystathionine is a key intermediate in the methionine metabolism and transsulfuration pathway. This accumulation indicates a redirection of sulfur flux toward glutathione biosynthesis to enhance cellular antioxidant defense [52]. This metabolic shift may reflect the prioritization of redox homeostasis. In contrast, compounds such as 2-O-caffeoyltartronic acid, 2-hydroxy-5-(trifluoromethoxy)benzoic acid, methylnorlichexanthone, and 4-nitrophenyl β-D-glucopyranosiduronic acid were commonly up-regulated in the 27 °C and 34 °C treatment groups. These compounds are mostly associated with phenylpropanoid metabolism and phenolic acid-related substances. Among them, 2-O-caffeoyltartronic acid possesses significant antioxidant activity [53,54], while methylnorlichexanthone is a common fungal secondary antimicrobial substance [55]. Their up-regulation suggests that at these higher temperatures, *G. lucidum* may enhance the synthesis of secondary metabolites by activating the phenylpropanoid metabolic pathway to cope with oxidative stress and microbial competition induced by elevated temperatures. Pathways coenriched across all temperatures, including signal transduction, carbohydrate metabolism, and biosynthesis of secondary metabolites, indicate a temperature independent core metabolic program, consistent with the conserved carbon metabolism of white-rot fungi [56].

### 4.4. Integrated Transcriptomic and Metabolomic Networks Revealing the Regulatory Network of Wheat Straw Fermentation by G. lucidum at Different Temperatures

Temperature significantly altered the correlation strength and clustering patterns between genes and metabolites. The gene and metabolite correlation network exhibited temperature-dependent features across three temperatures, while also maintaining conserved antagonistic relationships. Specifically, highly conserved antagonistic correlation patterns between phenylpropanoids or lignans and lipid or organic acid metabolism were maintained across all fermentation temperatures. This conserved antagonistic phenotype may reflect a fundamental metabolic trade-off in *G. lucidum* between efficient lignin degradation coupled with the accumulation of flavonoid and lignan secondary metabolites and maintenance of cell membrane homeostasis dependent on the lipid metabolite pool. Notably, *HSPC4* was expressed at all tested temperatures and showed correlations with metabolism of benzenoids, lipids, and organic acids. As a member of the heat shock protein family, which is known to be positively correlated with organism thermotolerance [57], *HSPC4* has also been reported to respond to cold stress [58]. *HSPC4* may additionally be involved in developmental processes, given that a previous study predicted a role for *HSP12*, *HSP71*, and *HSP60* in fruiting body formation of *F. filiformis* [59]. These observations collectively suggest that *HSPC4* may play a multifaceted role in both temperature stress response and developmental processes, a hypothesis that requires verification in future studies. This multifunctional role is analogous to that of *RL40A* and *RS27A*, which encode ubiquitin–ribosomal fusion proteins and are co-correlated with both lipid and flavonoid metabolites.

In this study, 3-oxoadipic acid and glycerophosphocholine were associated with multiple genes across temperatures and were therefore considered candidate metabolites related to organic acid and lipid metabolism, respectively. Glycerophosphocholine, a membrane lipid, which could be associated with signaling events that coordinate adaptive responses to stress [51,60], exhibited temperature-dependent gene association patterns in the present study. 3-oxoadipic acid, a putative intermediate in the intracellular catabolism of lignin-derived aromatic compounds [61], showed significant correlations with *CALM* and *COX2* at 27 °C, suggesting a potential association between aromatic carbon catabolism, calcium signaling, and mitochondrial respiration. These correlations indicate coordinated variation rather than direct causal or biosynthetic relationships. The exact molecular mechanisms need further study.

Beyond identifying 27 °C as the optimal temperature, the multi-omics data provide guidance for process optimization. The temperature-specific expression of *XYL2* (27 °C) and *PAPI* (34 °C), and suppression of *GUN3* and *FER1* (34 °C), offer candidate markers for monitoring degradation of cell wall components. The conserved negative correlation between phenylpropanoids and lipids suggests a manipulable metabolic trade-off. Moreover, the metabolic convergence between prolonged low-temperature and short-term high-temperature fermentation supports exploring multi-stage temperature strategies to balance lignin removal and cellulose preservation. It should also be noted that the strain used in this study (G5) is a specific isolate of *G. lucidum*, and its performance may not be representative of all strains of this species. Previous studies have reported substantial strain-to-strain variation in lignocellulose degradation selectivity among *G. lucidum* isolates [14]. While our strain exhibited higher cellulose loss, other isolates may achieve better cellulose preservation while maintaining efficient delignification. Therefore, screening a wider range of *G. lucidum* strains, using the temperature-response framework established here (particularly the optimal temperature of 27 °C) as a benchmark, represents a promising strategy for identifying superior candidates for feed-oriented straw bioprocessing.

## 5. Conclusions

Incubation temperature profoundly influenced both the extent and the selectivity of wheat straw conversion by *G. lucidum*. Fermentation at 27 °C and 34 °C led to greater degradation of lignocellulosic components than at 20 °C, with cellulose degradation remaining substantial, indicating limited selectivity for cellulose preservation. Despite differences in duration, prolonged low-temperature fermentation and shorter high-temperature fermentation showed partial convergence in their transcriptomic and metabolomic profiles, though in a nonlinear manner. The first two weeks were identified as the critical response window. Laccase and carbohydrate-active enzyme genes showed temperature-specific expression patterns. Conserved negative correlations between phenylpropanoid or lignan and lipid or organic acid metabolites, together with temperature-dependent remodeling of gene and metabolite networks, revealed distinct regulatory mechanisms underlying temperature modulated fungal straw conversion. These findings elucidate the molecular and biochemical basis of temperature-regulated fungal biomass conversion and provide a mechanistic foundation for optimizing *G. lucidum* based bioprocessing of straw into high-quality ruminant feed.

## Figures and Tables

**Table 1:** Cell wall composition of wheat straw fermented with *Ganoderma lucidum* at different temperatures for 0 to 6 weeks.

Temperature (°C)	Duration (W)	pH Value	Concentration (g kg^−1^ DM)				Absolute Amount (g)				DM Loss (%)
			TP	Cell	HC	ADL	TP	Cell	HC	ADL	
Control	0	6.12	28.1 ^d^	486.5	335.8 ^a^	58.4 ^a^	1.87 ^abc^	55.1 ^a^	22.5 ^a^	3.93 ^a^	0 ^f^
20	2	4.92	35.3 ^ab^	485.6	319.4 ^ab^	60.1 ^a^	2.20 ^a^	50.8 ^ab^	20.2 ^ab^	3.80 ^a^	5.77 ^e^
	4	4.7	35.8 ^a^	472.8	286.5 ^abcd^	47.3 ^ab^	2.20 ^a^	47.1 ^b^	17.8 ^bc^	2.93 ^ab^	7.40 ^de^
	6	4.58	33.3 ^abcd^	473.1	252.4 ^bcde^	48.6 ^ab^	1.97 ^ab^	43.2 ^bc^	15.0 ^cd^	2.87 ^ab^	11.01 ^d^
27	2	4.64	34.5 ^abc^	479.0	284.9 ^abcd^	59.0 ^a^	2.20 ^a^	48.3 ^ab^	18.0 ^de^	3.73 ^a^	5.57 ^e^
	4	4.38	30.1 ^bcd^	462.8	236.4 ^cde^	45.5 ^ab^	1.60 ^cd^	36.9 ^cde^	12.5 ^de^	2.40 ^bc^	21.10 ^c^
	6	4.46	33.7 ^abc^	484.9	194.9 ^e^	30.5 ^b^	1.57 ^cd^	33.3 ^e^	9.05 ^e^	1.43 ^c^	30.73 ^b^
34	2	4.58	29.5 ^cd^	474.6	317.4 ^abc^	48.6 ^ab^	1.73 ^bcd^	42.8 ^bcd^	18.7 ^ab^	2.87 ^ab^	11.07 ^d^
	4	4.54	32.9 ^abcd^	507.0	169.4 ^e^	41.3 ^ab^	1.57 ^cd^	35.0 ^ed^	8.00 ^e^	1.97 ^bc^	28.70 ^b^
	6	4.55	33.4 ^abcd^	477.8	208.7 ^de^	33.4 ^b^	1.43 ^d^	29.7 ^e^	9.03 ^e^	1.43 ^c^	35.2 ^a^
SEM		0.048	1.056	12.987	15.531	4.222	0.066	1.573	0.843	0.246	0.772
*p*-value											
Temperature		<0.0001	0.0269	0.6343	0.0032	0.0352	<0.0001	<0.0001	<0.0001	0.0003	<0.0001
Duration		<0.0001	<0.0001	0.8810	<0.0001	<0.0001	<0.0001	<0.0001	<0.0001	<0.0001	<0.0001
Temperature× Duration		0.0067	0.007	0.4214	0.0262	0.248	<0.0001	0.0028	0.0005	0.0399	<0.0001

Note: DM, dry matter; TP, true protein; Cell, cellulose; HC, hemicellulose; ADL, acid detergent lignin; SEM, standard error of the mean. Means with different superscripts within a column are significantly different based on two-way ANOVA followed by Tukey’s test (*p* < 0.05), *n* = 3.

## Data Availability

The original data presented in this study are openly available in the OMIX repository of the China National Center for Bioinformation (CNGB) under accession number OMIX018485 (associated with BioProject PRJCA068064). The raw sequence data reported in this paper have been deposited in the Genome Sequence Archive (GSA) at the National Genomics Data Center (NGDC), China National Center for Bioinformation/Beijing Institute of Genomics, Chinese Academy of Sciences, under accession number CRA046500, and are publicly accessible at https://ngdc.cncb.ac.cn/gsa, accessed on 1 May 2026.

## Supplementary Materials

The following supporting information can be downloaded at: https://www.mdpi.com/article/10.3390/jof12100730/s1, Table S1: KEGG pathway and Gene Ontology (GO) enrichment analyses of differentially expressed genes. Figure S1: orthogonal partial least squares discriminant analysis (OPLS-DA) score plot (A) and variable importance in projection (VIP) score plot (B) for metabolomic profiling.
