# Supplementary material for: Temperature-Dependent Conversion of Wheat Straw by Ganoderma lucidum: A Chemical and Multi-Omics Analysis
Source: J Fungi (Basel). 2026 Sep 30;12(10):730. doi: 10.3390/jof12100730 (PMC13705813; doi:10.3390/jof12100730)
Supplement: Supplementary file 1 [file jof-12-00730-s001.zip › Supplementary Figure S1.pdf]

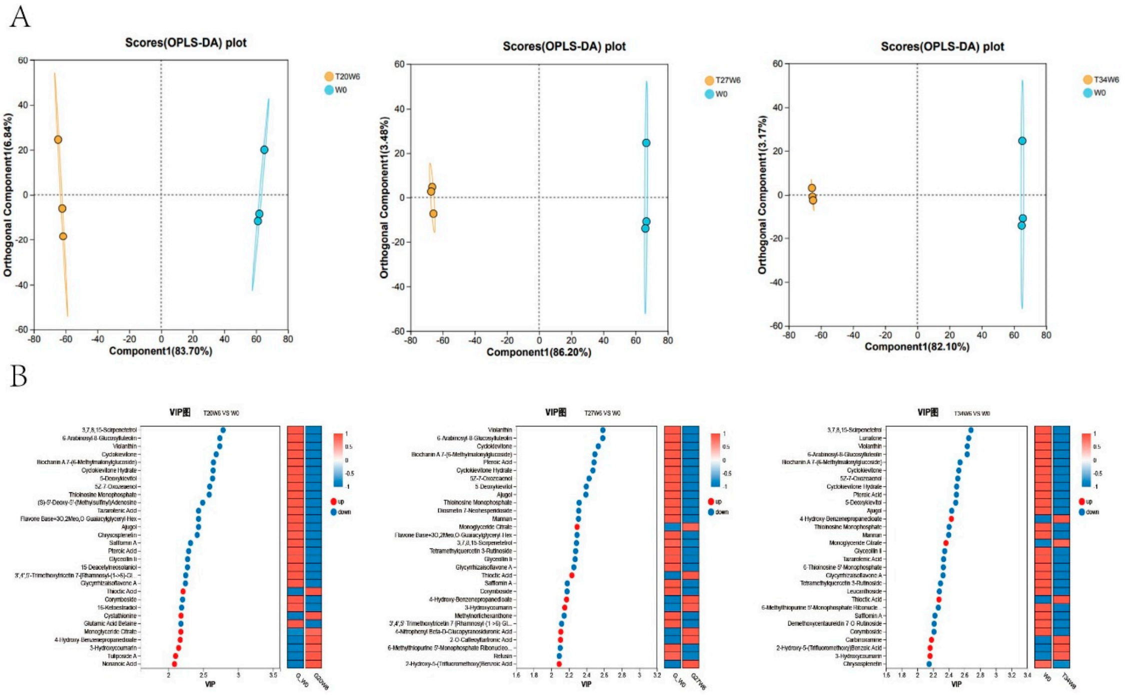

Figure S1: (A) Orthogonal Partial Least Squares Discriminant Analysis (OPLS-DA) score plot.(B) VIP score plot
